# Editorial: Cardiovascular immunometabolism - a complex interplay between the immune system and metabolism in the heart

**DOI:** 10.3389/fendo.2025.1613774

**Published:** 2025-04-30

**Authors:** Vikas Kumar, Gautham Yepuri, Gaurav Kumar, Ann Marie Schmidt

**Affiliations:** ^1^ Diabetes Research Program, Department of Medicine, New York University (NYU) Grossman School of Medicine, New York, NY, United States; ^2^ Department of Physiology, Medical College of Wisconsin, Milwaukee, WI, United States

**Keywords:** heart failure, immunometabolism, cuproptosis, extracellular vesicles (EVs), diabetes, and aging

Heart failure (HF) remains one of the leading causes of morbidity and mortality globally, posing a significant clinical and public health burden (1, 2). While traditionally conceptualized as a condition resulting from hemodynamic and structural dysfunction, HF is now increasingly understood as a complex, multifactorial syndrome (3, 4). It involves intricate interactions among genetic predispositions, environmental exposures, immune dysregulation, chronic inflammation, and metabolic remodeling (5, 6).

This Research Topic centers on immunometabolism, an emerging field that examines how immune responses are shaped by, and in turn shape, cellular metabolic processes. Growing evidence suggests that metabolic dysfunction within immune cells contributes to maladaptive cardiac remodeling and impaired tissue repair (7, 8). Key mechanisms include metabolic reprogramming in innate immune cells, the persistence of trained immunity, and aberrant inflammatory signaling (9). Another major focus is the role of extracellular vesicles (EVs)—membrane-bound particles secreted by virtually all cell types—as mediators of intercellular communication. EVs carry diverse bioactive cargos, including proteins, lipids, metabolites, and non-coding RNAs (e.g., miRNAs, lncRNAs, and circRNAs), and have been implicated in the modulation of immune responses, metabolic signaling, and cardiac remodeling processes in HF (10).

This Research Topic brings together a collection of articles exploring the interplay between immune cells, metabolism, EVs, and non-coding RNAs in the context of HF. These contributions provide valuable insights into the cellular and molecular underpinnings of HF and highlight novel diagnostic and therapeutic avenues.

## Key contributions

- Omoto et al. present a comprehensive review of the dual roles of EVs—both protective and pro-inflammatory—during cardiac injury. They explore how EVs modulate immune cell metabolism, particularly in the context of obesity and metabolic syndrome. The authors propose that EV cargo, including endocrine signals, miRNAs, lncRNAs, circRNAs, proteins, and metabolites, can act as upstream regulators of immunometabolic pathways. They also discuss how therapeutic loading of EVs could revolutionize treatment strategies in HF and related metabolic disorders.
- Tu et al. utilized machine learning and bioinformatics to re-analyze multiple microarray datasets, identifying a strong association between cuproptosis-related genes (CRGs) and the pathogenesis of HF. Their multivariable logistic regression models based on CRG expression suggest that cuproptosis modulates both metabolic and immune pathways in HF, offering potential diagnostic biomarkers and therapeutic targets.
- Chen et al. focused on diabetic heart failure, identifying four key CRGs (HSDL2, BCO2, CORIN, and SNORA80E) through bioinformatics analysis of GEO datasets. Among these, LOXL2 was most significantly linked to diabetic HF. The study also constructed an lncRNA–miRNA–mRNA network and predicted MECOM as a key transcription factor driving cuproptosis in cardiomyocytes, advancing our understanding of HF pathogenesis in diabetic patients.
- Huang et al. examined factors contributing to accelerated aging in patients with type 2 diabetes mellitus (T2DM) and coronary heart disease (CHD). By analyzing clinical data from 216 patients, they developed phenotypic age acceleration (PhenoAgeAccel) metrics and identified several risk factors, including neutrophil count, urea, adenosine deaminase, and TyG index, while cholinesterase was identified as a protective factor. These findings underscore the clinical significance of aging-related biomarkers in T2DM-associated HF.
- Tian et al. investigated the prognostic impact of stress hyperglycemia in critically ill patients with cardiogenic shock using the MIMIC-IV database. Stratifying patients based on stress hyperglycemia ratio (SHR), they found that elevated SHR was associated with longer mechanical ventilation, extended ICU and hospital stays, and increased mortality. Their analysis suggests SHR may serve as a valuable prognostic indicator in this high-risk population.

Collectively, these contributions illuminate the complex interplay between immune responses, metabolic alterations, non-coding RNAs, and extracellular vesicle signaling in the pathophysiology of heart failure. They also underscore the translational potential of targeting these pathways to develop novel diagnostics and therapeutic strategies.

We anticipate that this Research Topic will serve as a comprehensive and insightful resource for researchers investigating the latest advances in immunometabolism and its therapeutic implications in cardiovascular disease.
